# Predicting BRICS NIFTY50 returns using XAI and S.A.F.E AI lens

**DOI:** 10.3389/frai.2025.1668700

**Published:** 2025-09-18

**Authors:** Indranil Ghosh, Tamal Datta Chaudhuri, Golnoosh Babaei, Paolo Giudici, Emanuela Raffinetti

**Affiliations:** ^1^IT & Analytics Area, Institute of Management Technology Hyderabad, Hyderabad, Telangana, India; ^2^Bengal Economic Association, Kolkata, India; ^3^Department of Economics and Management, Universita di Pavia, Pavia, Italy

**Keywords:** global funds, BRICS, transnational volatility, SHAP-based XAI, S.A.F.E AI

## Abstract

**Purpose:**

Global fund managers, in their effort toward risk diversification and generating higher returns, design portfolios that consist of financial assets of various countries. In the process, they expose their investors not only to the fundamentals of the assets but also to transnational volatility, macroeconomic shocks of different countries, and exchange rate fluctuations. These factors make forecasting returns from such global funds quite difficult and, at the same time, challenging. To aid global fund managers and investors, this study presents a forecasting framework for predicting returns from Goldman Sachs BRICs Nifty 50 Developed Markets Index (BRICS NIFTY 50), which is a traded and listed financial asset. It is a global portfolio, which not only exposes investors to the fundamentals of different companies but also to country risk.

**Design, methodology, and approach:**

Gradient boosting regression (GBR) and SHAP-based XAI are used to identify the top significant country-specific explanatory variables. Subsequently, with the selected variables, GBR, CatBoost, Light Gradient Boosting Machine (LGBM), Extreme Gradient Boosting (XGBoost), Random Forest (RF), and Extra Tree Regressor (ETR) are applied for forecasting returns from BRICS NIFTY 50. Along with standard evaluation tools, the S.A.F.E AI framework is used for measuring predictive accuracy, sustainability, and contribution of each predictor. To evaluate the relative efficacy of the six predictive models, the underlying research resorts to a multi-criteria decision-making (MCDM) framework.

**Findings:**

We find that country-specific market volatility, industrial performance, financial sector development, and exchange rate fluctuations explain global returns significantly. Furthermore, the exercise also reveals that explanatory factors specific to India, China, and Brazil emerge to be relatively important.

**Research limitations and implications:**

The study focuses on a single index. Future work will extend it to other indices and global funds.

**Practical implications:**

The proposed methodology will be of practical use to global fund managers and investors. Policymakers may find it useful for identifying factors that make foreign direct investment and portfolio investment attractive.

**Originality and value:**

Development of a two-step forecasting framework, identifying effects of country-specific explanatory variables, and applying different evaluation criteria to measure predictive efficiency underscore the novelty of the work.

## 1 Introduction

BRICS economies include five existing members, namely, Brazil, Russia, India, China, and South Africa, as well as Egypt, Ethiopia, Iran, Saudi Arabia, and the UAE, who have joined in January 2024 or have been invited (El-Aal et al., 2025; Hasan et al., 2023; Khare et al., 2023; Sethi et al., 2025). According to Azevedo et al. (2024), “These 10 nations account for around 40% of both crude oil production and exports. They also account for one-quarter of global GDP, two-fifths of global trade in goods, and nearly half of the world's population.” In view of their importance as a group, being a major producer and consumer of energy, along with trade relations, infrastructure financing, and technological cooperation, they have been a focal point of discussion. Khalfaoui et al. (2023) found that BRICS have generated high average stock returns and have low correlations with those of the developed markets. It is thus quite natural that global funds operate within the BRICS economies. One such example is Templeton BRIC Fund. There are ETFs and indices that are designed around the BRICS economies. This study focuses on Goldman Sachs BRICs Nifty 50 Developed Markets Index (BRICS NIFTY 50), which is composed of 50 listed companies with market capitalization greater than $1 billion, which are incorporated in Europe, the USA, Japan, Korea, and Australia and have significant exposure to BRICS and other emerging economies. It is a traded instrument. There are global mutual funds available in India including Invesco India—Invesco Global Equity Income FoF Fund Direct—Growth, Aditya Birla Sun Life Global Excellence Equity FoF Fund Direct—Growth, and ICICI Prudential Global Advantage Fund (FOF) Direct—Growth.

BRICS NIFTY 50 may be sensitive to transnational volatility and macroeconomic shocks internal to the BRICS countries, along with exchange rate fluctuations, financial market maturity, and the rate of economic growth. Thus, investor returns from this instrument would not only be sensitive to the fundamental performance of the companies but also to the overall economic environment of these economies. It would be interesting to perform a time series analysis of the index returns and determine which of the countries in the index have influenced it most. This study develops an explainable AI-based forecasting framework for predicting returns from this index, which is akin to forecasting portfolio/fund returns. Global fund managers need to be cautious about such instruments as they are sensitive to financial crises that can rapidly spread across borders (Reinhart and Rogoff, 2009). Furthermore, they are also dependent on the domestic financial sector and fiscal policies. According to Kaminsky et al. (2003), financial markets are sensitive to whether governments will defend their exchange rate and also to the financial fragility of the banks and financial institutions. Alakkari et al. (2022) underscored the importance of macroeconomic and uncertainty factors in predicting and understanding the volatility of market sentiment.

This study advances an integrated AI framework for predicting returns from this global index. Taking into consideration various factors mentioned in the literature, the explanatory variables would consist of country-specific stock market volatility, the macroeconomic state represented by industrial growth and services sector growth, and also exchange rate volatility explaining trade relations and government intervention for currency stability. All the variables included in the analysis and their definition are elaborated in a later section.

To accomplish the research endeavors, initially, the major features explaining movement in daily returns of the chosen index are identified using a combined framework of gradient boosting regression (GBR) and Shapley (SHAP) value-inspired explainable artificial intelligence (XAI) approaches. These explanatory variables are then used to train five additional ensemble machine learning algorithms, namely, CatBoost, Light Gradient Boosting Machine (LGBM), Extreme Gradient Boosting (XGBoost), Random Forest (RF), and Extra Tree Regressor (ETR), in addition to GBR, for prediction purposes. Afterward, we resort to the S.A.F.E AI framework, introduced by Giudici et al. (2024), to holistically comprehend the feature impact on the predictive process in terms of sustainability, accuracy, and explainability aspects. Finally, the metrics corresponding to the S.A.F.E AI model and other predictive indicators are used in a multi-criteria decision making (MCDM) framework to rank the methods. For this, MEREC and combined compromise solution (CoCoSo) tools are used.

The major contributions of the present work are as follows. First, the effects of transnational volatility, economic growth, and trade relations on returns from a global fund are ascertained. Toward this end, appropriate explanatory variables are identified. Second, XAI methodology is used for automatic feature selection, and this leads to deeper insights into the model. Third, the model reveals the relative importance of the BRICS countries in the movement of the global index returns. This will be useful for global fund managers. Fourth, the model identifies the factors within the countries that drive their attractiveness as an investment destination and hence can guide policymakers. Finally, the deployment of S.A.F.E AI for evaluating predictive efficiency is a novel addition to conventional efficiency metrics used in the literature.

The remaining part of the study proceeds as follows. The survey of past literature is enunciated in Section 2 to emphasize the existing research gaps and to rationalize the positioning of the present study. Section 3 describes the variables in detail. The different methodological components are explained in Section 4. The findings of the research are discussed in detail in Section 5, focusing on the three key agendas: predictive outcome, feature contribution, and model explanation. The key implications of the research findings are outlined in Section 6. Section 7 concludes the study with major observations, current scope and limitations, and future research direction.

## 2 Past research

This section elicits the review of pertinent research into two segments. The first segment enunciates the trend and highlights the literature meant for analyzing global stock indices, while the other segment focuses on the methodological progress of XAI and S.A.F.E. AI on financial market modeling.

### 2.1 Global stock index modeling and forecasting:

Ali et al. (2024) exemplified the utility of two neural network architectures, multilayer perceptron and radial basis function, in accurately predicting and classifying stock prices amidst economic policy uncertainty. Ayadi et al. (2025) examine the impact of macroeconomic and COVID-19 news-linked indicators on Latin American stock indexes. The results suggest that macroeconomic indicators of the United States, associated with monetary policy and real activity, induced a stronger effect on returns and volatility than domestic news-related ones. Cohen and Aiche (2025) used an ensemble of different neural network models, namely, a feedforward neural network (FNN), a long short-term memory (LSTM) network, and a gated recurrent unit (GRU) network, for accurately predicting bitcoin price using a series of technical indicators, macroeconomic variables, and Google trends. The findings of the research duly rationalize the efficacy of the framework in enabling accurate trading in the market. Manasseh et al. (2025) established strong interlinkage between stock market prospect and agricultural sector development in Nigeria and South Africa through the lens of dynamic autoregressive distributed lag model. Overall findings indicated that policies to promote stock market development positively boosted agricultural development in both countries. Qin et al. (2025) developed a novel CMDMamba model, inspired by the Mamba architecture of state-space models (SSMs), integrated with a dual convolutional feedforward network (DconvFFN) framework for accurately capturing price fluctuations at the micro- and macro-levels in the financial market. The hybrid model outperformed several benchmark forecasting architectures in predicting four global stock indices. Wan et al. (2024) examined the dynamic return and volatility connectedness of global ESG stock indexes using a time-varying parameter vector autoregression (TVP-VAR) framework to enable joint time–frequency analysis. The findings suggested that Europe and North America-linked ESG stock indexes experienced outward spillovers, whereas the Asia-Pacific and Indian counterparts tilted to inward spillovers. Return and volatility connectedness were primarily concentrated at high-frequency and low-frequency components, respectively. Research by Helmi et al. (2025) evidenced the presence of pronounced cross-moment spillovers among MENA stock markets. Implied volatility and geopolitical risk were deemed to causally impact the spillover dynamics in specific regimes. Throughout the literature, close co-movement and interlinkage dynamics connecting global stock indexes, economic growth, commodity futures responsible for trade development, and foreign exchange rates have been documented in the literature (Ahelegbey and Giudici, 2022; Belcaid, 2024; Cadena-Silva et al., 2025; Chen and Zhang, 2023; Peng and Yang, 2025).

The recent predictive analytics research on global financial markets has seen renewed interest in machine and deep learning methodologies designed to estimate highly accurate forecasts (Ferro et al., 2024; Lee and Anderl, 2025; Liu et al., 2025; Magner et al., 2025). Basher and Sadorsky (2025) deployed Random Forests, boosting, extremely randomized trees, and support vector machines for predicting clean energy stock prices. Different technical indicators appeared highly impactful for predicting stock prices of solar, wind, and nuclear energy sectors. Gkonis and Tsakalos (2025) exploited the golden Jackal optimizer algorithm for fine-tuning several standard deep neural network architectures for predicting stock prices. The utility of the metaheuristic in fetching highly accurate forecasts was duly rationalized. Gülmez (2025) combined attention-based feature selection with genetic algorithm-optimized fuzzy systems for precisely predicting stock prices in short- and long-range intervals. The framework outperformed standalone machine and deep learning models. Lin and Hsu (2024) underlined the utility of fuzzy set theoretic approaches in augmenting the predictive capability of machine learning algorithms in forecasting Taiwan biotech stock index. Potential advantage of incorporating the power of large language models (LLMs) to gauge the impact of conventional and social media buzz on financial markets has also been argued (Jadhav and Mirza, 2025).

Scrutiny of the above-mentioned strand of literature emphasizes on the need and relevance of close examination of empirical patterns of the global stock indices for reaping diversification benefits, identifying investment opportunities, etc. The predictive analytics literature of the same primarily relies upon autoregressive structures using lagged values and technical indicators solely. To unveil practical implications in parallel to contributing toward the methodological front, it is important to integrate macroeconomic indicators in conjunction with lagged information-based technical indicators for developing forecasting frameworks. In addition, effective assessment of macroeconomic constructs in the prediction process can facilitate trading. The underlying research aims to bridge the gap.

### 2.2 XAI and S.A.F.E AI for financial variables

XAI methodologies have become increasingly significant in financial market analyses, offering transparency and interpretability to complex AI models used in this domain. Various tools of XAI frameworks, namely, SHAP, local interpretable model-agnostic explanations (LIME), partial dependence plots (PDP), and accumulated local effects (ALE) plots, have been reported to be successful in explaining the high-end machine and deep learning workflows (Adam, 2024; Babaei et al., 2023; Bussmann et al., 2025; Ghallabi et al., 2024; Ghosh et al., 2022; Ghosh and De, 2024; Lu and Lin, 2024). Teplova et al. (2023) leveraged SHAP-driven XAI on top of neural network models for identifying key determinants of stock liquidity in the Russian market during the COVID-19 pandemic. It was observed that resource use served an integral role during the pandemic. Navier-Stokes equation-driven stock price momentum forecasting framework proposed by Ghosh and Chaudhuri (2022) was interpreted through SHAP-based XAI methodology. The findings implied non-uniformity in feature contribution across different sectors. Muhammad et al. (2024) engaged SHAP-based global feature importance and LIME-based local feature contribution inspection assessment to interpret the machine learning-driven stock trend prediction process. The insights were useful for risk mitigation and strategic interventions. The utilities of the said methodologies in cross-sectional financial data, i.e., bankruptcy prediction and loan default assessment, have also been acknowledged in the literature (Kadkhoda and Amiri, 2025; Monje et al., 2025). The conceptualization of S.A.F.E AI to measure the risk quotient of AI systems manifested through sustainability, accuracy, fairness, and explainability was initially proposed by Giudici et al. (2024) for decoding the performance of regression models, classification trees, ensemble methods, and neural networks in modeling financial variables. Subsequently, Giudici et al. (2024) utilized Shapley-Lorenz values for estimating explainability, accuracy, fairness, and sustainability scores to evaluate performance of several variants of deep neural networks, autoregressive neural network, long short-term memory network (LSTM), and gated recurrent units (GRU) networks to predict US Dollar-Euro rate, US Dollar-Chinese Yuan rate, SP500 index, and Gold and crude oil futures. The framework emerged to be extremely efficient in uncovering patterns pertinent to the interaction of lagged-based predictor variables. Babaei et al. (2025) further exemplified the utility of the S.A.F.E AI framework in resolving standard data classification tasks.

Thus, the ongoing surge in traction toward XAI methodologies in relevant literature is imminent owing to their utmost efficacy in interpreting contributions of predictor variables in driving complex predictive frameworks. Meanwhile, the emergence of S.A.F.E AI in manifesting the critical dimensions of the machine and deep learning algorithms in executing any predictive task facilitates extracting holistic insights on the extent of explainability globally and locally across predictors, ethical issues, and sustainability in addition to gauging accuracy, as well. Hence, leveraging the integrated approach of XAI and S.A.F.E AI becomes imperative to demystify the temporal dynamics of BRICS NIFTY 50.

## 3 Data and variables

**Figure 1:**
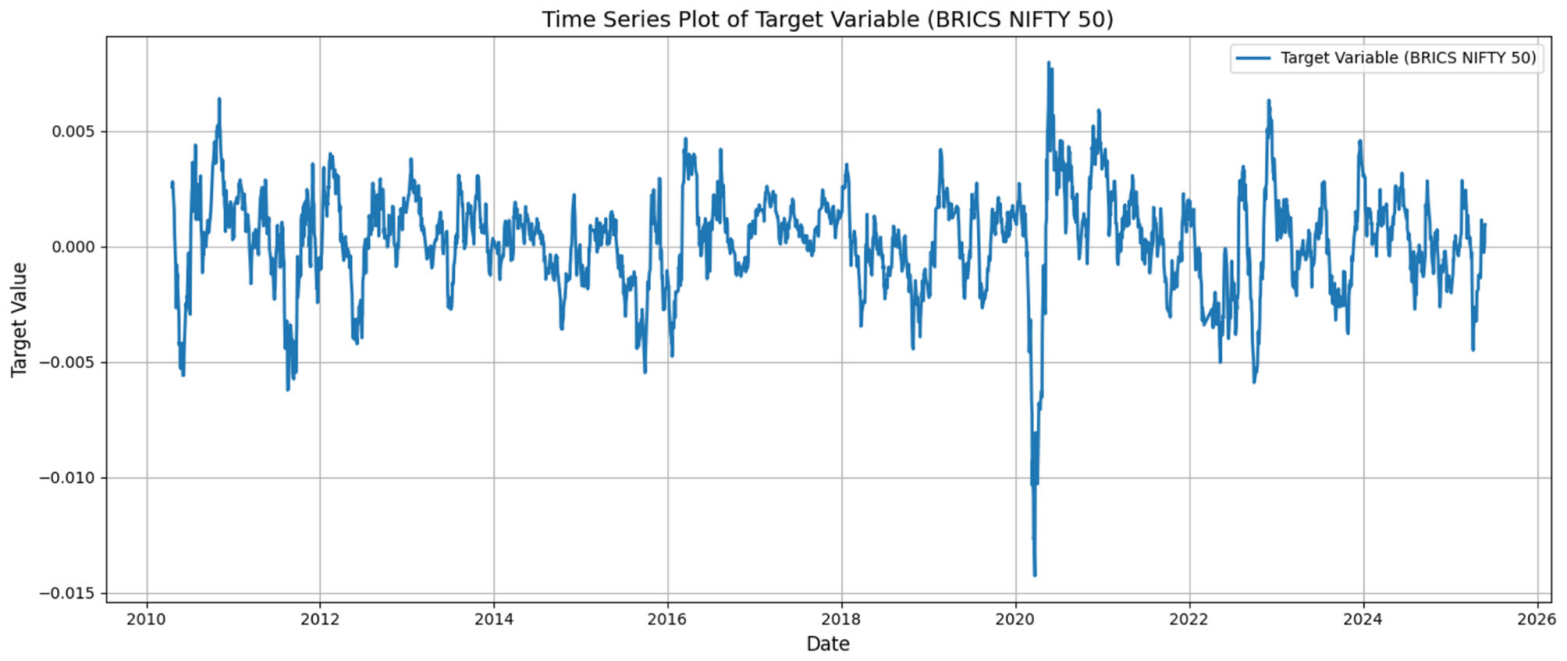
Daily returns of BRICS NIFTY 50 index.

The daily closing prices of the BRICS NIFTY 50 index have been compiled for examination for a time period of 25 February 2010 to 28 May 2025. The Bloomberg data repository is used for data collection. The iterative imputer class from the “scikit-learn” library is employed to address the issue of missing values present in the dataset. In the realm of statistics, a prevalent technique for imputing absent values entails modeling each feature as a function of other existing variables in a Round-Robin fashion. We have utilized the “IterativeImputer” class from the Python-based “scikit-learn” library, in which missing values are estimated in accordance with the foundational research conducted by Little and Rubin (2019). The daily returns of NIFTY BRICS 50 are computed using Equation 1 to transform the same as the target variable.


(1) $$\begin{array}{l} Return_{t}^{BRICSNIFTY50}=\frac{P_{t}-P_{t-1}}{P_{t-1}} \end{array}$$

where *P*_*t*_ represents the closing price at day *t*. Figure 1 depicts the temporal pattern of the time series variable.

The occasional spikes and random movements are imminent from mere visualization. We further introspect the key statistical properties of the variable, which are outlined in Table 1.

**Table 1:** Key statistical properties of the target variable.

**Properties**	**NIFTY BRICS 50**
Minimum	−0.0911
Maximum	0.0874
Mean	0.0003
Median	0.0006
SD	0.0113
Skewness	−0.3000
Kurtosis	7.2203
Shapiro Test	0.9281^***^
AD Test	37.246^***^
ADF Test	−36.7388^***^
Terasvirta's NN Test	878.89^***^
Hurst Exponent	0.5094

^***^Significant at 1% significance level, SD, standard deviation; AD, Anderson-Darling; SW, Shapiro–Wilk; ADF, Augmented Dickey-Fuller; NN, neural network.

The empirical characteristics of the BRICS NIFTY 50 series clearly indicate that the underlying target construct does not follow a normal distribution as apparent from the Shapiro and AD test outcome. As the variable represents the daily return series, the same has prevailed to be stationary as expected, as apparent from the ADF test statistic. The outcome of Terasvirta's NN test suggests the presence of steep non-linear patterns in daily returns of NIFTY BRICS 50 is apparent. The said aspect of the target variable rationalizes the deployment of ensemble machine learning algorithms for predictive analysis. Finally, the value of the Hurst exponent is close to 0.5, which theoretically testifies a time series as a pure random walk. Thus, the close resemblance of the chosen series with Brownian motion makes modeling and interpretation tasks arduous. The non-linear traits and the extent of randomness of the chosen variable rationalize the utilization of the advanced research methodologies, which are non-parametric and invariant to multicollinearity.

As discussed, the five categories of explanatory variables used in this research to explain and predict the daily closing returns of the NIFTY BRICS 50 index are enunciated in Table 2.

**Table 2:** Details of explanatory variables.

**Category of variables**	**Features**	**Data repository**
Stock market volatility indicators	Historic Volatility of BOVESPA (BOVESPAHV) Index (Brazil), Historic Volatility of MOEX (MOEXHV) Index (Russia), Historic Volatility of NIFTY (NIFTYHV) Index (India), Historic Volatility of CSI (CSIHV) Index (China), Historic Volatility of JSE (JSEHV) Index (South Africa)	Bloomberg Data Repository, Official Portal of Nifty Indices, www.investing.com
Industrial growth indicators	Returns of Basic Materials (IMAT) Index (Brazil), Returns of MOEX Metal (MOEXMET) Index (Russia), Returns of NIFTY Manufacturing (NMFG) Index (India), Returns of CSI Industrial (CSI800) Index (China), Returns of JSE 25 Industrial (JSE25) Index (South Africa)	Bloomberg Data Repository, Official Portal of Nifty Indices, www.investing.com
Service growth indicators	Returns of Consumer Stock (ICON) Index Brazil, Returns of MOEX Consumer (MOEXCON) Index (Russia), Returns of NIFTY Services (NSERV) Index (India), Returns of CSI Consumer Services (CSICON) Index (China), Returns of JSE Top 40 (JSE40) Index (South Africa)	Bloomberg Data Repository, Official Portal of Nifty Indices, www.investing.com
Financial growth indicators	Returns of Financial (IFIN) Index (Brazil), Returns of MOEX Financials (MOEXFIN) Index (Russia), Returns of NIFTY Financial Services (NFIN) Index (India), Returns of CSI Financial (CSIFIN) Index, Returns of JSE Financial 15 (JSE15) Index (South Africa)	Bloomberg Data Repository, Official Portal of Nifty Indices, www.investing.com
Foreign exchange rate	United States Dollar—Brazilian Real (USDBRL) Rate, United States Dollar—Russian Ruble (USDRBL) Rate, United States Dollar—Indian Rupee (USDINR) Rate, United States Dollar—Chinese Yuan (USDCNY) Rate, United States Dollar—South African Rand (USDZAR) Rate	Bloomberg Data Repository, Official Portal of Nifty Indices, www.investing.com
Lagged indicators	One-Day Lagged Value BRICS NIFTY Returns (LAG1), Two-Day Lagged Value BRICS NIFTY Returns (LAG2), Three-Day Lagged Value BRICS NIFTY Returns (LAG3), Four-Day Lagged Value BRICS NIFTY Returns (LAG4), Five-Day Lagged Value BRICS NIFTY Returns (LAG5)	Bloomberg Data Repository

**Figure 2:**
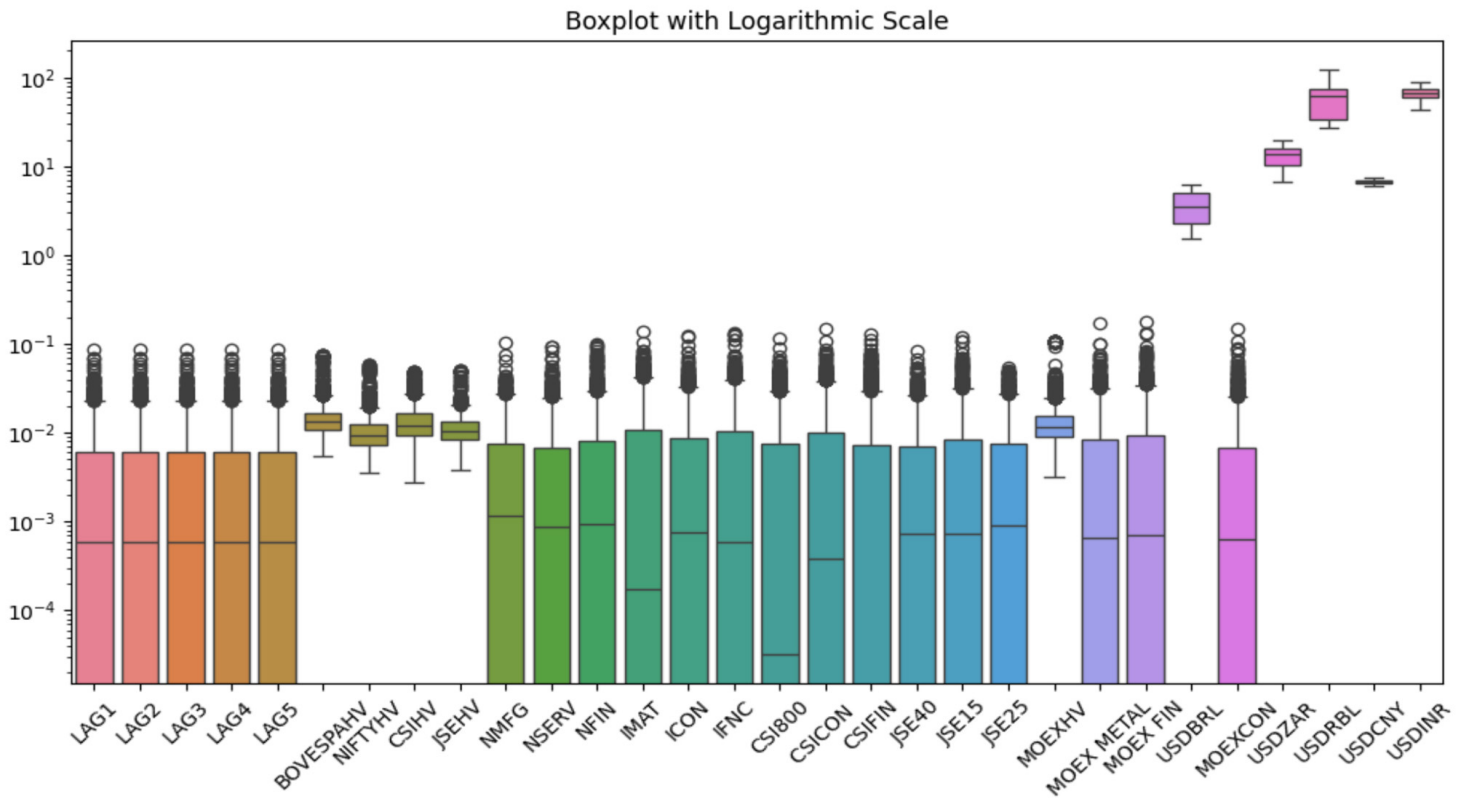
Boxplot of explanatory variables.

The historic volatility of the corresponding stock market volatility indicators is calculated by estimating the rolling 20-day standard deviations of returns of the respective indices, which are computed using Equation 1. The Bloomberg data repository is used to collect data on indices linked to Brazil, China, and South Africa, while Russia-linked features are collated from the portal www.investing.com. The daily observations of India-centric variables are compiled from the official portal of Nifty Indices (www.niftyindices.com). Figure 2 depicts the boxplot of the explanatory indicators on a logarithmic scale.

## 4 Methodology

**Figure 3:**
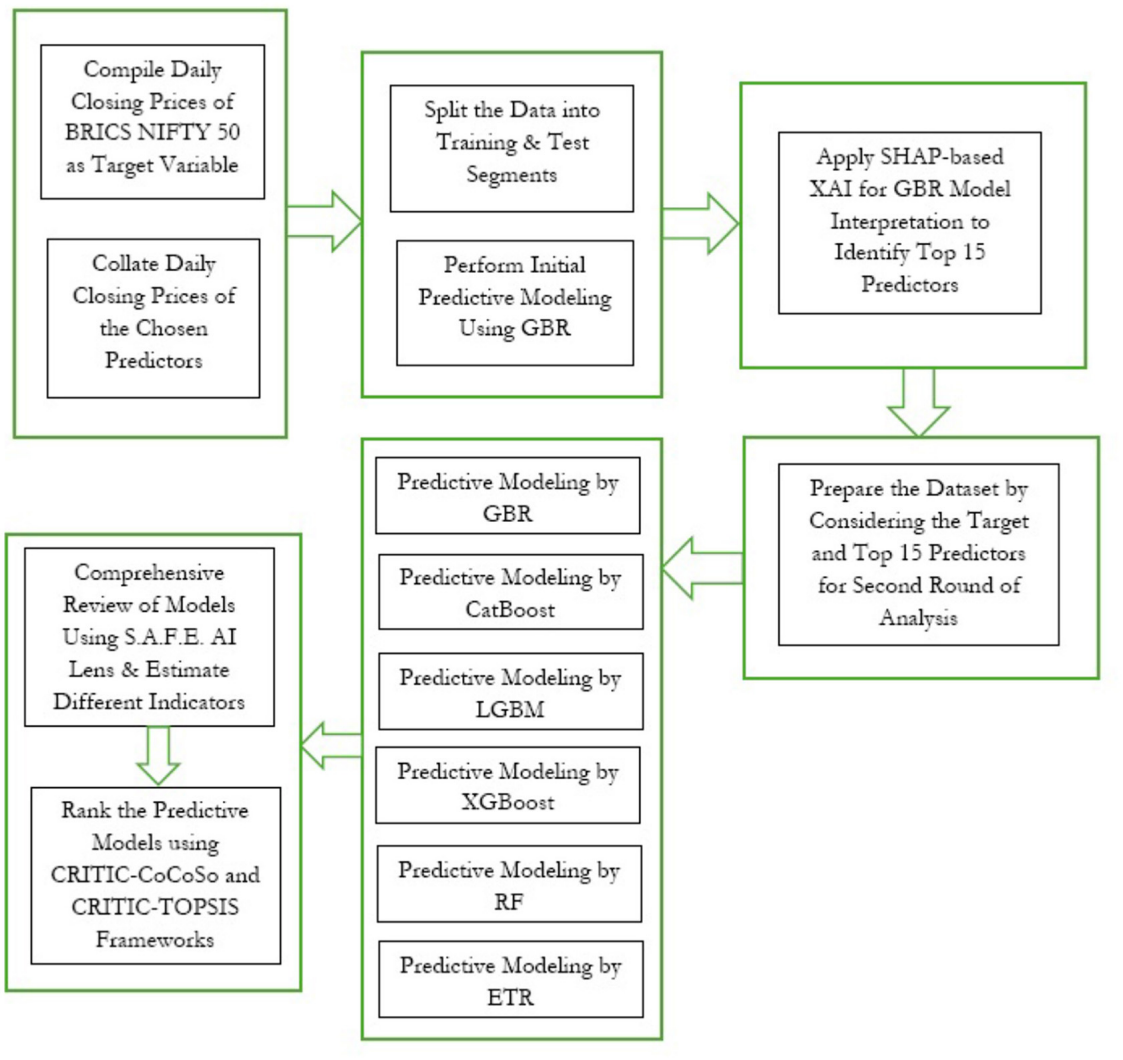
Integrated research framework.

In this section, we thoroughly elucidate the explainable AI framework adopted in this study to accomplish the key research endeavors. Figure 3 visually depicts the integrated approach.

To identify and interpret the impact of the chosen explanatory variables in explaining the variability of the target construct, combined usage of GBR and SHAP-driven XAI is invoked initially. The prime endeavor of this first stage of predictive analysis is not to gauge the accuracy of prediction but to critically assess the significant explanatory features. We now describe the details of both methodologies.

### 4.1 Gradient boosting regression (GBR)

Introduced by Schapire and Singer (1999), gradient boosted regression (GBR) is an ensemble machine-learning technique that constructs predictions using a forward stage-wise approach. It builds upon the traditional boosting framework, enhancing it by optimizing the model based on gradient-derived error rates. At each iteration, a regression tree serves as the base learner, trained sequentially to minimize the residuals of the preceding model. This step-by-step ensemble learning process significantly contributes to improving prediction accuracy by systematically correcting errors from previous stages.

Mathematically, for a response variable (*y*) and a set of explanatory constructs (*x*), GBR strives to obtain an approximate $\overset{\sim }{F}\left(x\right)$ of the function, *F*(*x*) binding response, and input variables by optimizing the loss function, *L*(*y, F*(*x*)). Equation 2 summarizes the process.


(2) $$\begin{array}{l} \overset{\sim }{F}\left(x\right)={argmin}_{F\left(x\right)}L_{y,x}\left(y,F\left(x\right)\right) \end{array}$$

The conventional squared sum of residual is used as the loss function. The gradient of the loss function is determined as follows:


(3) $$\begin{array}{l} {\overset{\sim }{y}}_{i}=-{\left[\frac{\partial L\left(y_{i},F\left(x_{i}\right)\right)}{\partial F\left(x_{i}\right)}\right]}_{F\left(x\right)=F_{m-1}\left(x\right)},i=1,2,\ldots ,N. \end{array}$$

The estimation process is generalized as regression trees, *h*(*x*_*i*_; *a*) with parameter *a* as weak learners. The parameters are computed as follows:


(4) $$\begin{array}{l} a_{m}=argmin_{\alpha ,\beta }\overset{N}{\underset{i=1}{\sum }}{\left[{\overset{\sim }{y}}_{i}-\beta h\left(x_{i};a\right)\right]}^{2} \end{array}$$

where *a*_*m*_ refers to the parameters obtained at m^th^ iteration, and β represents the weight value of respective weak learners. The practical implementation of GBR is simulated in a Python programming environment by applying the “*scikit-learn*” library with 500 decision trees for regression.

### 4.2 SHAP-based feature selection

On top of the fitted GBR model, the methodological framework employs the SHAP-driven XAI inspection to unveil the critical predictor variables. The SHAP measure provides new directions to estimate the contribution of individual features in the prediction process (Lundberg and Lee, 2017). Mathematically, it is computed using Equation 5:


(5) $$\begin{array}{l} \emptyset_{i}=\underset{S\subseteq N\left\{i\right\}\backslash \left\{i\right\}}{\sum }\frac{|S|!\left(n-|S|-1\right)!}{n!}\left[v\left(S\cup \left\{i\right\}\right)-v\left(S\right)\right]\; \end{array}$$

where ∅_*i*_ denotes the contribution of the *i*^*th*^ feature, N is the set of all features with cardinality n, S is the subset of *N* excluding feature *i*, and *v(S)* is the predicted outcome using the feature set S. The explanation is specified by applying Equation 6:


(6) $$g\left(z\prime \right)=\emptyset_{0}+\sum_{j=1}^{M}\emptyset_{j}z_{j}^{\prime }$$

where *z*′∈{0, 1}^*M*^, and *M* denotes the number of features under consideration.

The predictor variables can conveniently be ranked on the basis of their respective relative contributions as gauged by the mean SHAP values. Highly effective and successful utilization of SHAP in unveiling the feature contributions in predictive analytics of financial time series modeling problems has been duly reported in the literature (Ghosh et al., 2022). As the primary objective of utilizing XAI in this work is to identify the most relevant explanatory variables, the SHAP approach is invoked. For a local level inspection, the LIME model could be used, while a thorough comprehension of any explanatory variables could be gauged through ALE or PDP plots in the future. In this work, we identify the top 15 predictors from the list of predictors defined in Table 2. As discussed, the second iteration of predictive modeling using five different ensemble methodologies, barring the GBR model, is carried out subsequently using the select explanatory features with strong predictive influence for drawing final insights on predictions and deeper inspection through the S.A.F.E. AI lens. In the second iteration of predictive modeling, delving into the accuracy of generating forecasts is underscored. We now thoroughly outline key principles of the other ensemble models used to meet the endeavors.

### 4.3 CatBoost

It is a modification of the standard gradient boosting method for enabling predictive analysis in the presence of both categorical and numeric explanatory variables (Dorogush et al., 2017). It incorporates the principle of ordered boosting and induces multiple permutations of the dataset to estimate gradients. Its base learners are made of oblivious trees, which improves the stability of the model. The framework is tailored to fetch predictions of supreme precision, avoiding overfitting while ensuring interpretability. The base learners are used in a sequential manner and are driven by calculating the gradient loss functions for individual observations. The objective function integrates a loss function and regularization term as


(7) $$\begin{array}{l} Objective=\overset{n}{\underset{i=1}{\sum }}L\left(y_{i},{\hat{y}}_{i}\right)+\overset{K}{\underset{k=1}{\sum }}\Omega \left(f_{k}\right) \end{array}$$

where *L* accounts for the loss function reflecting prediction accuracy, *y*_*i*_, and ŷ_*i*_ refer to actual and predicted figures for the *i*^*th*^ sample, *K* is the number of trees, and Ω(*f*_*k*_) denotes the regularization term. The final prediction is obtained by scaling the aggregate outcome of all trees using the leaning rate (η).


(8) $$\begin{array}{l} F\left(x\right)=\overset{K}{\underset{k=1}{\sum }}\eta f_{k}\left(x\right) \end{array}$$

CatBoost leverages the ordered boosting for tree pruning and optimizing the tree architecture to minimize loss. The “*catboost*” Python library has been used for simulation using 500 base learners.

### 4.4 Light Gradient Boosting Machine (LGBM)

The LGBM (Ke et al., 2017) is an extended version of gradient boosting and is a highly efficient and scalable gradient boosting model typically used in complex predictive modeling. It is driven by decision tree ensembles and is optimized for high-speed performance, lower memory usage, and superior accuracy, particularly on large and complex datasets. The procedural steps of LGBM comprise histogram-based splitting, leaf-wise tree growth, and gradient-based one-side sampling for training the regression tree-base learners for sequential ensemble processing. Rather than evaluating every possible split point, LightGBM discretizes continuous features into binned histograms and evaluates accuracy accordingly, which significantly accelerates training on large datasets. The leaf-wise growth strategy selects the leaf with the maximum loss reduction at each iteration and is responsible for enhancing the prediction accuracy while keeping computational costs reasonably low. The said approach also ensures a reduction in overfitting compared to the level-wise growth of regression trees. The objective function and the philosophy of drawing the final prediction in LGBM are similar to that of CatBoost procedure as expressed in Equations 7 and 8. Alternatively, the model prediction in iteration t is updated as


(9) $$\begin{array}{l} {\hat{y}}^{\left(t\right)}={\hat{y}}^{\left(t-1\right)}+\eta \cdot h^{\left(t\right)}\left(x\right) \end{array}$$

where ŷ^(*t*)^ denotes the LGBM outcome after *t*^*th*^ iteration, *h*^(*t*)^(*x*) is the newly fitted decision tree for regression, and η refers to the learning rate. It also deploys gradient-driven tree pruning to eliminate redundant branches and nodes from the tree structure. The practical implementation is carried out using the “*lightgbm*” Python package with 500 regression trees.

### 4.5 Extreme Gradient Boosting (XGBoost)

It is one of the variants of classical Boosting algorithms for machine learning (Chen and Guestrin, 2016). Its basis lies in the ensemble-based machine learning mechanism to obtain final predictions, using a series of basic learners in a sequence to overcome the weaknesses of individual learners while obtaining final outputs. The XGBoost model imitates the Boost tree model, which incorporates Taylor's second-order expansion in the form of a loss function. Therefore, XGBoost is more universal and offers better performance in ML applications. The XGBoost model is immune to overload and multicollinearity of the dataset. Mathematically, the framework constructs the K number of regression trees in sequence to facilitate the training process of individual trees using residual components of previous trees. This process leads to the creation of new trees with lower residuals and contributes to the accuracy of the design. Each ensemble model performs the sum of K functions for predicting $\overset{ˆ}{y_{i}}$.


(10) $$\begin{array}{l} \overset{ˆ}{y_{i}}=F\left(X_{i}\right)=\overset{K}{\underset{k=1}{\sum }}f_{k}\left(X_{i}\right),f_{k}\in F,i=1,\ldots n\; \end{array}$$

where *F* is the representation of the function space of the regression tree model, and *f*_*k*_(.) refers to an independent CART model. The loss function (ℓ) of the XGBoost model is represented as follows:


(11) $$ℒ=\sum_{i=1}^{n}\ell \left(\gamma_{i}{\overset{⌢}{\gamma }}_{i}\right)+\sum_{k=1}^{K}\eta (f_{d})$$

where ℓ(.) measures the deviation between γ_*i*_ (actual) and $\overset{ˆ}{\gamma_{i}}$ (prediction), and $\eta =\mu T+\frac{1}{2}\lambda |\left|\omega \right|{|}_{2}^{2}$ is the regularization parameter. The “xgboost” Python package is used for implementing the model with 500 regression trees.

### 4.6 Random Forest (RF)

It is a widely used ensemble learning algorithm introduced by Breiman (2001), designed to improve the predictive performance of decision trees through bootstrap aggregation (bagging) and random feature selection. It is a robust, non-parametric method particularly well-suited to non-linear, high-dimensional, and noisy regression tasks. Mathematically, for a training data segment, be $D={\left\{(x_{i},y_{i})\right\}}_{i=1}^{n}$, with inputs $x_{i}\in {\mathbb{R}}^{p}$ and targets *y*_*i*_∈ℝ, RF grows an ensemble of *M* regression trees {*T*_1_, *T*_2_, …, *T*_*M*_} via the following procedure:

Bootstrap Sampling: For each tree *T*_*m*_, a bootstrap sample $D_{m}\subset D$ of size *n* is drawn with replacement.

Optimal Feature Selection: At each split node within a tree, a random subset of *k* ≤ *p* features is considered for splitting.

Optimal Split Selection: For the selected subset, the split point is chosen to minimize the impurity, often using the variance of the target variable in regression:


(12) $$ℐ(t)=\frac{1}{\left|t\right|}\sum_{i\in t}{\left(y_{i}-y_{t}\right)}^{2}$$

The best feature-threshold pair (f^*^, t^*^) is the one that minimizes the weighted impurity of the child nodes. Each tree *T*_*m*_ generates a prediction ŷ_*m*_(*x*) for input *x*. The final output of the Random Forest is the average of all tree predictions:


(13) $$\begin{array}{l} {\hat{y}}_{RF}\left(x\right)=\frac{1}{M}\overset{M}{\underset{m=1}{\sum }}{\hat{y}}_{m}\left(x\right) \end{array}$$

This ensemble averaging leads to variance reduction. The “*sklearn*” utility using Python programming has been leveraged to implement the RF model with 500 regression trees.

### 4.7 Extra Tree Regressor (ETR)

Introduced by Geurts et al. (2006), ETR is an extension of the conventional RF model for facilitating ensemble learning. The ETR technique deploys base learners in the form of a series of unpruned regression trees constructed by applying a traditional top-down methodology. The said approach is different from the RF framework, which generally relies upon a two-step operation involving bagging and bootstrapping, growing the base learners for eventual regression analysis. The ETR model resorts to a deterministic splitting method, wherein instead of using a random subset of explanatory features for the branching operations in base learners, the entire feature set is evaluated to identify the optimal splitting candidate. The split point of each feature is, however, selected randomly. Let *S*(*f, t*) denotes the variance reduction from splitting feature *f* at threshold *t*. Then, the best split is


(14) $$(f^{*},t^{*})=argmax_{\left(f,t\right)}S(f,t)\;$$

Each individual tree *T*_*m*_ yields a prediction ŷ_*m*_(*x*) for an input *x*. The final prediction is obtained by averaging over all trees:


(15) $$\begin{array}{l} {\hat{y}}_{ETR}\left(x\right)=\frac{1}{M}\overset{M}{\underset{m=1}{\sum }}{\hat{y}}_{m}\left(x\right) \end{array}$$

The averaging reduces variance and enhances generalization. The “*sklearn*” utility in Python programming environment is utilized to simulate the ERT predictive structure with 500 base estimators.

### 4.8 Performance evaluation

To evaluate the predictive modeling, four measures, namely, mean absolute error (MAE), mean squared error (MSE), root mean squared error (RMSE), and index of agreement (IA), are used. They are computed as follows:


(16) $$\begin{array}{l} MSE=\;\frac{1}{N}\overset{N}{\underset{t=1}{\sum }}{\left(y_{t}-{\hat{y}}_{t}\right)}^{2}\; \end{array}$$

(17) $$\begin{array}{l} RMSE=\sqrt{\frac{1}{N}\overset{N}{\underset{t=1}{\sum }}{\left(y_{t}-{\hat{y}}_{t}\right)}^{2}}\; \end{array}$$

(18) $$\begin{array}{l} MAE=\frac{1}{N}\overset{N}{\underset{t=1}{\sum }}|y_{t}-{\hat{y}}_{t}|\; \end{array}$$

(19) $$IA=1-\frac{\sum_{t=1}^{N}{\left(y_{t}-{\hat{y}}_{t}\right)}^{2}}{\sum_{t=1}^{N}{\left\{\left|{\hat{y}}_{t}-y\right|+|y_{t}-y|\right\}}^{2}}\;$$

where *y*_*t*_ and ŷ_*t*_ refer to the actual and predicted observations, and *y* is the average of actual observations. The values of MSE, RMSE, and MAE should ideally be as low as possible, while IA figures should be close to 1 to indicate quality predictions.

### 4.9 S.A.F.E AI

To systematically monitor the compliance of AI-driven modeling holistically, Giudici et al. (2024) proposed S.A.F.E AI framework, which embodies four key principles to unveil deeper insights, namely, “S” for sustainability, “A” for accuracy, “F” for fairness, and “E” for explainability. Decoding predictive modeling through the lens of S.A.F.E AI framework offers meaningful and actionable insights of capital significance. Babaei et al. (2025) subsequently advanced the rank graduation box by providing appropriate manifest indicators to account for each of the dimensions. The rank graduation accuracy (RGA) metric corresponds to the overall accuracy, implying the precision of the obtained predictions by the respective ensemble machine learning model. Sustainability in the context of the robustness of the predictive model can be gauged by the rank graduation robustness (RGR) indicator. Fairness, on the other hand, typically reflects the impact of different population groups on prediction models, is applicable for categorical variables, and is measured by the rank graduation fairness indicator (RGF). As the present research does not include any categorical variables in the structured methodology, the said indicator is not used in this study. Finally, the contributions of the explanatory variables are tracked by rank graduation explainability (RGE) scores, which are derived on the basis of the Shapley-Lorenz value, a variant of the SHAP methodology. The mathematical bedrock of RGA, RGR, and RGE is jotted down below.

#### 4.9.1 Rank graduation accuracy (RGA)

The RGA quantifies the predictive accuracy of the underlying model. It aims to gauge the precision of the forecasts by monitoring the alignment of the predicted values $\overset{ˆ}{Y}$ with the observed values *Y* manifested through rank concordance. It is constructed by comparing the cumulative distribution of ranks in the predicted vs. actual values using a concordance curve, which lies between the Lorenz and dual Lorenz curves.

Let y^*^ be the vector of observed values; y^**^ represents vector of predicted values, *n* is the sample size, $y^{*}$ is the mean of y^*^, $r_{i}^{*}$ refers to the non-decreasing ranks of y^*^, and $r_{n+1-i}^{*}$ is the non-increasing ranks of y^*^. The rank graduation accuracy is then estimated as


(20) $$RGA=\frac{\sum_{i=1}^{n}\left\{\frac{1}{ny^{*}}\left(\sum_{j=1}^{i}y_{r_{n+1-j}^{*}}^{*}-\sum_{j=1}^{i}y_{r_{j}^{**}}^{*}\right)\right\}}{\sum_{i=1}^{n}\left\{\frac{1}{ny^{*}}\left(\sum_{j=1}^{i}y_{r_{n+1-j}^{*}}^{*}-\sum_{j=1}^{i}y_{r_{j}^{*}}^{*}\right)\right\}}$$

The numerator represents the area between the concordance curve and the dual Lorenz curve, and the denominator corresponds to the total area between the Lorenz and dual Lorenz curves. RGA values close to 1 infer sound concordance between predictions and true values, implying accurate predictive performance.

#### 4.9.2 Rank graduation robustness (RGR)

The RGR metric is meant to ascertain the sustainability of a model when subject to random perturbations in the input data. This is essential for evaluating AI reliability under adversarial or noisy conditions. It is calculated as follows:


(21) $$RGR=\frac{\sum_{i=1}^{n}\left\{\frac{1}{ny^{*}}\left(\sum_{j=1}^{i}y_{r_{n+1-j}^{*}}^{*}-\sum_{j=1}^{i}{\hat{y}}_{r_{j}^{p}}^{p}\right)\right\}}{\sum_{i=1}^{n}\left\{\frac{1}{ny^{*}}\left(\sum_{j=1}^{i}y_{r_{n+1-j}^{*}}^{*}-\sum_{j=1}^{i}{\hat{y}}^{p}\right)\right\}}$$

where ŷ^*p*^ refers to the prediction on perturbed data, and *r*^*p*^ is the corresponding ranking. Higher values of RGR, i.e., close to 1, testify to the robustness of the model.

#### 4.9.3 Rank graduation explainability (RGE)

The RGE quantifies the contribution of any predictor variable (*x*_*k*_) to the overall prediction process by comparing the full model with a reduced model that excludes the chosen predictor. It is determined as:


(22) $$RGE=1-\;\frac{\sum_{i=1}^{n}\left\{\frac{1}{ny^{*}}\left(\sum_{j=1}^{i}y_{r_{n+1-j}^{*}}^{*}-\sum_{j=1}^{i}{\hat{y}}_{r_{j}}^{-x_{k}}\right)\right\}}{\sum_{i=1}^{n}\left\{\frac{1}{ny^{*}}\left(\sum_{j=1}^{i}y_{r_{n+1-j}^{*}}^{*}-\sum_{j=1}^{i}y_{r_{j}^{*}}^{*}\right)\right\}}$$

where ${\hat{y}}^{{-x}_{k}}$ accounts for predicted values from the reduced model. Higher RGE values reflect stronger predictive influence. The contributions of individual explanatory variables can be aggregated to indicate the overall explainability of the entire model. The “*safeai*” package developed by (Babaei and Giudici 2025) in the Python programming environment is utilized to estimate these figures for respective predictive models.

### 4.10 Model ranking

To evaluate the relative efficacy of the six predictive models, the underlying research resorts to a multi-criteria decision-making (MCDM) framework for accomplishing the task. The present study integrates the method based on the removal effects of criteria (MEREC) (Keshavarz-Ghorabaee et al., 2021), a tool to evaluate criteria weights with a combined compromise solution (CoCoSo) (Yazdani et al., 2019) and technique for order preference by similarity to ideal solution (TOPSIS) (Hwang and Yoon, 1981), as tools for ranking alternatives. The MEREC method is first applied to determine objective weights of the criteria reflecting model predictive and S.A.F.E AI indicators, as manifested by MSE, RMSE, MAE, IA, RGA, RGR, and RGE. Then, the six ensemble models, namely, GBR, CatBoost, LGBM, XGBoost, RF, and ETR, are evaluated by CoCoSo and TOPSIS models separately by obtaining the preference score and closeness coefficient, respectively. The principles of these tools are as follows.

#### 4.10.1 Method based on the removal effects of criteria (MEREC)

The procedural steps of the MEREC method are jotted down as follows.

Step 1: Given *m* alternatives evaluated over *n* criteria, the decision matrix *X* is defined as follows:


(23) $$\begin{array}{l} X=\left[\begin{array}{cccc} x_{11} & x_{12} & \ldots & x_{1n}\\ x_{21} & x_{22} & \ldots & x_{2n}\\ \ddots \\ x_{m1} & x_{m2} & \ldots & x_{mn} \end{array}\right] \end{array}$$

where *x*_*ij*_ represents the evaluation value of alternative *i* with respect to criterion *j*.

Step 2: The decision matrix is normalized by converting each element *x*_*ij*_ to *n*_*ij*_ based on benefit (*B*) or cost (*C*) criteria as follows:


(24) $$n_{ij}=\{\begin{array}{cc} \frac{\min_{k}x_{kj}}{x_{ij}}, & ifj\in B\\ \frac{x_{ij}}{\max_{k}x_{kj}}, & ifj\in C \end{array}$$

Step 3: The overall performance score (*S*_*i*_) of the alternatives is estimated using a logarithmic aggregation function as follows:


(25) $$\begin{array}{l} S_{i}=ln\left(1+\frac{1}{n}\overset{n}{\underset{j=1}{\sum }}|ln\left(n_{ij}\right)|\right) \end{array}$$

This transformation ensures that lower normalized values contribute more significantly.

Step 4: The performance after criterion Removal (*S*^′^_*ij*_) for each criterion *j* is calculated by computing the performance of each alternative *i* by excluding criterion *j*:


(26) $$S_{ij}^{\prime }=ln\left(1+\frac{1}{n-1}\sum_{\begin{array}{l} k=1\\ k\neq j \end{array}}^{n}\left|ln(n_{ik})\right|\right)$$

Step 5: The removal effect (*E*_*j*_), representing the total deviation caused by removing criterion *j* across all alternatives, is determined as follows:


(27) $$E_{j}=\sum_{i=1}^{m}|S_{ij}^{'}-S_{i}|$$

Step 6: The final weights (*w*_*j*_) of the criteria are computed by normalizing the removal effects.


(28) $$\begin{array}{l} w_{j}=\frac{E_{j}}{\overset{n}{\underset{k=1}{\sum }}E_{k}} \end{array}$$

#### 4.10.2 Combined compromise solution (CoCoSo)

The estimated weights by the MEREC method are directly used in the CoCoSo framework, applying the following steps.

Step 1: The decision matrix *X* is initially constructed.


(29) $$\begin{array}{l} X=\left[\begin{array}{cccc} x_{11} & x_{12} & \ldots & x_{1n}\\ x_{21} & x_{22} & \ldots & x_{2n}\\ \ddots \\ x_{m1} & x_{m2} & \ldots & x_{mn} \end{array}\right] \end{array}$$

where *x*_*ij*_ reflects the performance of alternative I, when rated against the criterion *j*, and *m* and *n* denote the number of alternatives and criteria, respectively.

Step 2: The normalization of the decision matrix is obtained by applying Equations 30 and 31 for benefit and criteria, respectively.


(30) $$\begin{array}{l} r_{ij}=\frac{x_{ij}-x_{ij}}{x_{ij}-x_{ij}};\text{for benefit type criteria } \end{array}$$

(31) $$\begin{array}{l} r_{ij}=\frac{x_{ij}-x_{ij}}{x_{ij}-x_{ij}};\text{for cost type criteria} \end{array}$$

Step 3: The weighted sum of the comparability sequence (*S*_*i*_) and the whole power weight of comparability sequences (*P*_*i*_) corresponding to the alternatives are then estimated using gray relational generation approach and WASPAS multiplicative attitude, respectively, as follows:


(32) $$\begin{array}{l} S_{i}=\overset{n}{\underset{j=1}{\sum }}\left(w_{j}r_{ij}\right)\; \end{array}$$

(33) $$\begin{array}{l} P_{i}=\overset{n}{\underset{j=1}{\sum }}{\left(r_{ij}\right)}^{w_{j}}\; \end{array}$$

Step 4: The relative weights of the individual alternatives are then derived by applying and aggregating three appraisal score strategies as follows:


(34) $$\begin{array}{r} k_{ia}=\frac{P_{i}+S_{i}}{\overset{m}{\underset{i=1}{\sum }}\left(P_{i}+S_{i}\right)} \end{array}$$

(35) $$\begin{array}{r} k_{ib}=\frac{S_{i}}{min_{i}S_{i}}+\frac{P_{i}}{min_{i}P_{i}} \end{array}$$

(36) $$\begin{array}{r} k_{ic}=\frac{\lambda \left(S_{i}\right)+\left(1-\lambda \right)\left(P_{i}\right)}{\left(\lambda \;max_{i}S_{i}+\left(1-\lambda \right)min_{i}P_{i}\right)};0\leq \lambda \leq 1 \end{array}$$

In this work, we have considered λ = 0.5.

Step 4: The final ranking is obtained by computing the aggregate score (*k*_*i*_) as follows:


(37) $$\begin{array}{l} k_{i}={\left(k_{ia}k_{ib}k_{ic}\right)}^{\frac{1}{3}}+\frac{1}{3}\left(k_{ia}+k_{ib}+k_{ic}\right) \end{array}$$

Higher value of *k*_*i*_ implies higher position of the alternative in relative ranking spectrum.

#### 4.10.3 Technique for order preference by similarity to ideal solution (TOPSIS)

Proposed by Hwang and Yoon (1981), the method is grounded in the concept that the chosen alternative should have the shortest distance from the positive ideal solution (PIS) and the farthest distance from the negative ideal solution (NIS). The steps are outlined below:

Step 1: The decision matrix, as defined for CoCoSo method, is normalized by invoking vector normalization on each element (*x*_*ij*_).


(38) $$\begin{array}{l} r_{ij}=\frac{x_{ij}}{\sqrt{\overset{m}{\underset{i=1}{\sum }}x_{ij}^{2}}}\forall i,j\; \end{array}$$

Step 2: The weighted normalized matrix is obtained using weights (*w*_*j*_) estimated by MEREC method as follows:


(39) $$\begin{array}{l} v_{ij}=w_{j}\cdot r_{ij}\forall i,j\; \end{array}$$

Step 3: The positive ideal solution (PIS) and negative ideal solutions (NIS) are marked as follows:


(40) $$\begin{array}{l} A^{+}=\left\{\underset{i}{\max}v_{ij}|j\in B;\underset{i}{\min}v_{ij}|j\in C\right\}\; \end{array}$$

(41) $$\begin{array}{l} A^{-}=\left\{\underset{i}{\min}v_{ij}|j\in B;\underset{i}{\max}v_{ij}|j\in C\right\}\; \end{array}$$

where $A^{+}=\left\{v_{1}^{+},v_{2}^{+},...,v_{n}^{+}\right\}$ and $A^{-}=\left\{v_{1}^{-},v_{2}^{-},...,v_{n}^{-}\right\}$ represent the PIS and NIS, respectively.

Step 4: The degree of dissimilarity of the underlying alternatives the PIS $\left(S_{i}^{+}\right)$ and NIS $\left(S_{i}^{-}\right)$ are computed by applying the conventional Euclidean norm as follows:


(42) $$\begin{array}{l} S_{i}^{+}=\sqrt{\overset{n}{\underset{j=1}{\sum }}{\left(v_{ij}-v_{j}^{+}\right)}^{2}}\; \end{array}$$

(43) $$\begin{array}{l} S_{i}^{-}=\sqrt{\overset{n}{\underset{j=1}{\sum }}{\left(v_{ij}-v_{j}^{-}\right)}^{2}}\; \end{array}$$

Step 5: The relative closeness coefficient (*C*_*i*_) to obtain the final ranking is calculated as follows:


(44) $$\begin{array}{l} C_{i}=\frac{S_{i}^{-}}{S_{i}^{+}+S_{i}^{-}}\; \end{array}$$

where 0 ≤ *C*_*i*_ ≤ 1. A higher value of *C*_*i*_ indicates that the alternative is closer to the ideal solution and, hence, more preferable.

The “*pymcdm*” library has been used in the Python programming environment to execute the combined approach of MEREC-CoCoSo and MEREC-TOPSIS checks to rank the six different predictive frameworks.

## 5 Results and discussions

In this section, we chronologically present the findings of different steps, as depicted in Figure 3, to elaborate on the research findings.

### 5.1 Outcome of initial modeling

**Figure 4:**
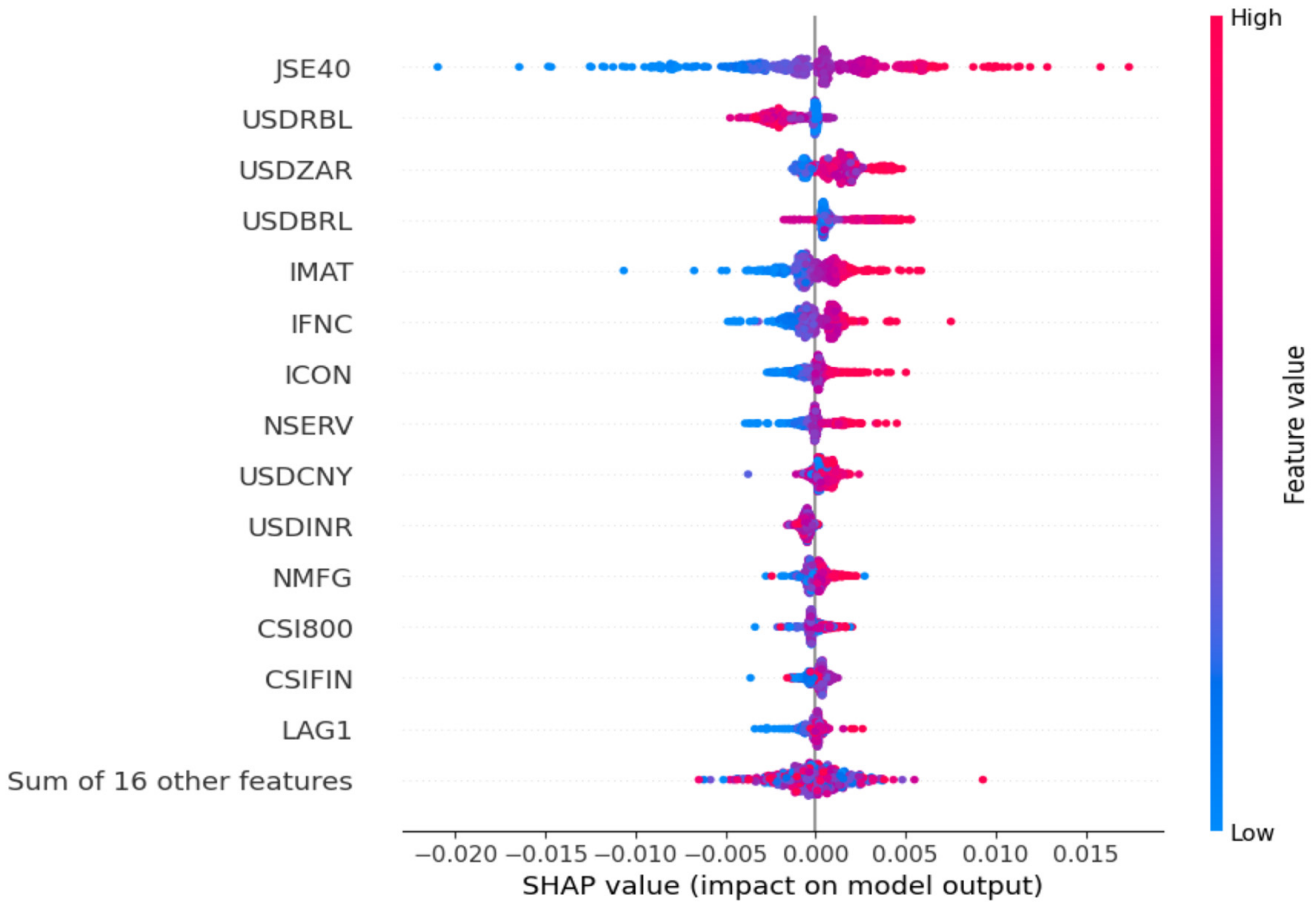
Feature contribution assessment by SHAP.

At first, the GBR model is trained on the dataset to uncover the critical predictors explaining the target variable, represented by the daily returns of the BRICS NIFTY 50 index. The dataset is partitioned into in-sample (80%) and out-of-sample (20%) subsets for conducting predictive analytics. The partition has been forward-looking, which has been reported as an ideal setup for predictive analytics financial time series (Jana et al., 2021; Ghosh and De, 2024). The in-sample segment is used to train the model, while the out-of-sample segment evaluates the efficacy of the trained models on test data points. The SHAP values characterizing the influence of the respective explanatory features are computed on the test data segment on top of the GBR model. The initial setup uncovers the top 15 explanatory features that largely drive the prediction process. Figure 4 illuminates the outcome of the global feature importance assessment by SHAP methodology.

The JSE40, USDRBL, USDZAR, USDBRL, and IMAT emerge to be the top five explanatory features for demystifying the evolutionary patterns of the BRICS NIFTY 50 returns. Several sectoral indices of India and China, namely, NSERV, NMFG, CSI800, and CSIFIN, also exert significant predictive influence. Barring USDRBL, no Russia-linked variables are deemed to feature in the top 15 significant feature list. Similarly, except for JSE40, no other South Africa-linked variables reside in the feature list. Hence, an inference can be drawn that explanatory features from Brazil, India, and China are relatively more prudent in anticipating the future trends of the target financial variable than Russian and South African counterparts. As an autoregressive feature, LAG1 resembles the 15th spot. Relatively low reliance on lagged-based indicators conforms with the random walk behavior of the time series, as marked by the Hurst exponent mentioned in Table 1. The prominence of trade relations in the form of the select foreign exchange rates in driving the prediction process is apparent. The absence of any volatility indicators in the top 15 feature list suggests the immunity of the target variable toward uncertainty and fear in the chosen markets. Mostly, the higher values of the features are linked with higher contributions.

### 5.2 Applied predictive modeling

The second round of predictive exercises is built on the top 15 explanatory features by GBR, CatBoost, LGBM, XGBoost, RF, and ETR models. Similar to the initial predictive modeling setting, the training (80%) and test (20%) segments are created using a forward-looking partition to facilitate the exercises. Table 3 reflects upon the overall predictive performance of the test data segment.

**Table 3:** Predictive performance on test segment.

**Models**	**MSE**	**RMSE**	**MAE**	**IA**
GBR	0.0001	0.0100	0.0078	0.7058
CatBoost	0.0001	0.0083	0.0063	0.7943
LGBM	0.0001	0.0090	0.0069	0.7538
XGBoost	0.0001	0.0090	0.0069	0.7456
RF	0.0001	0.0084	0.0064	0.7800
ERT	0.0001	0.0081	0.0061	0.7792

**Figure 5:**
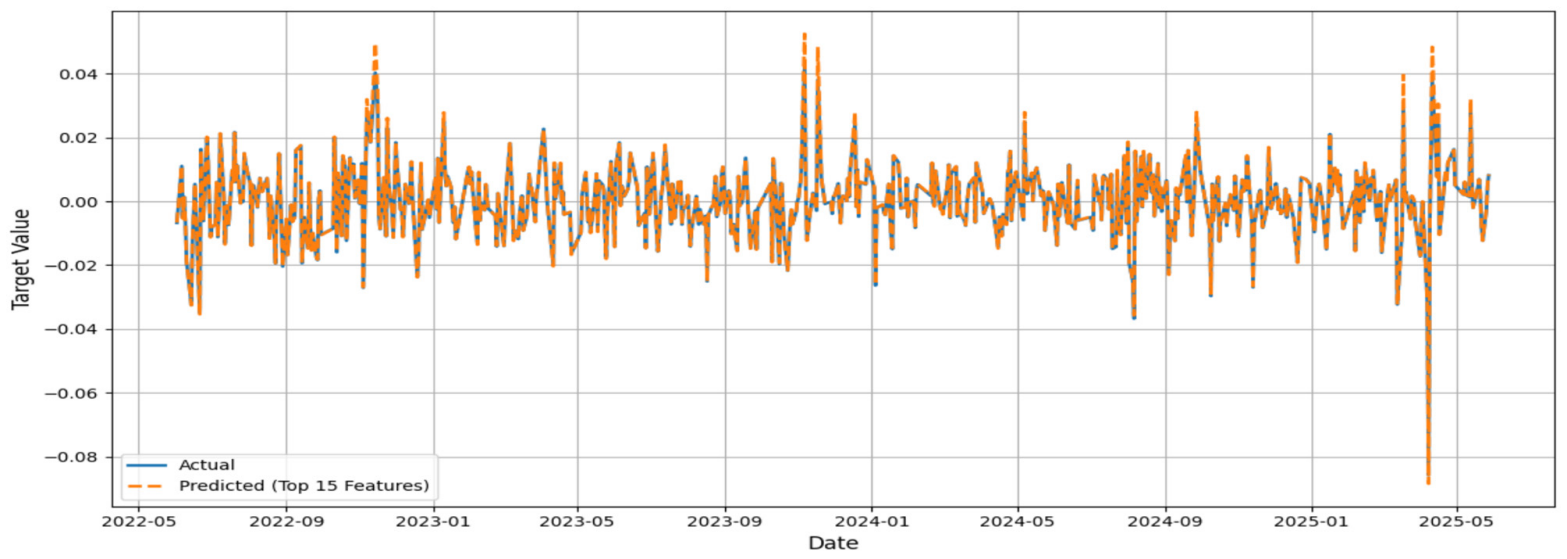
Actual and predicted figures for GBR modeling.

**Figure 6:**
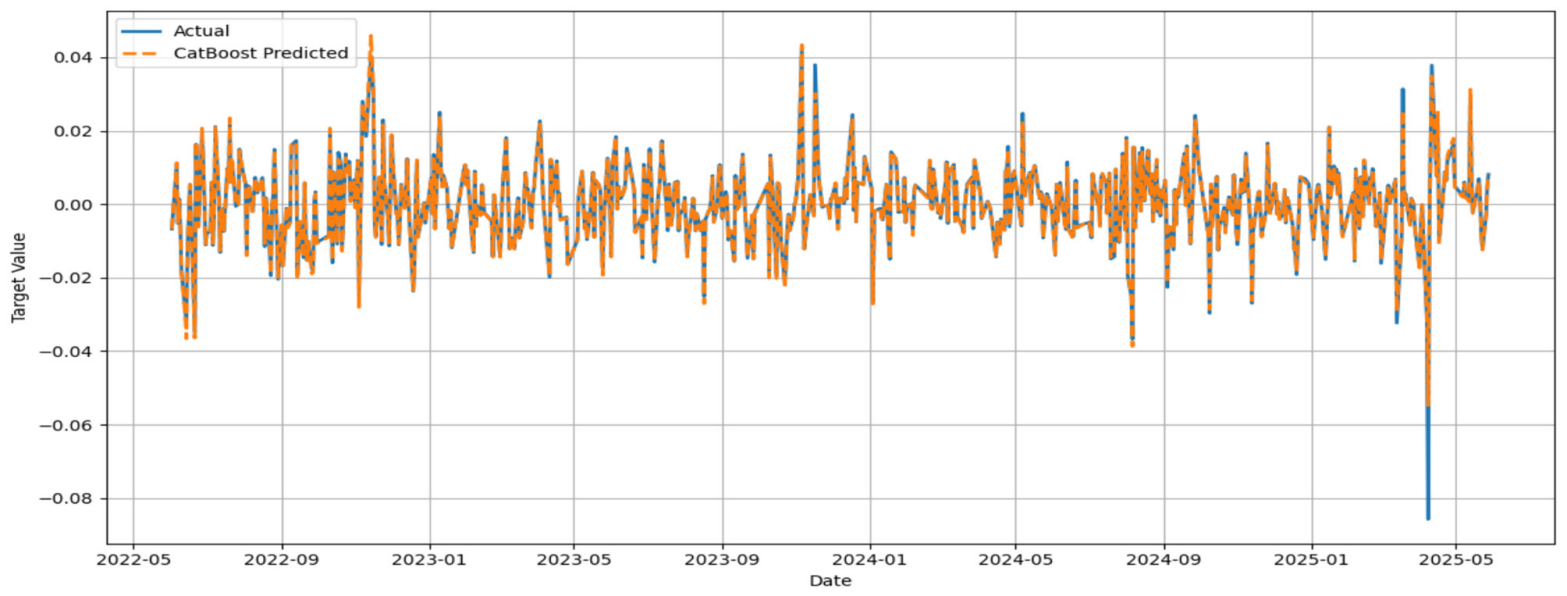
Actual and predicted figures for CatBoost modeling.

**Figure 7:**
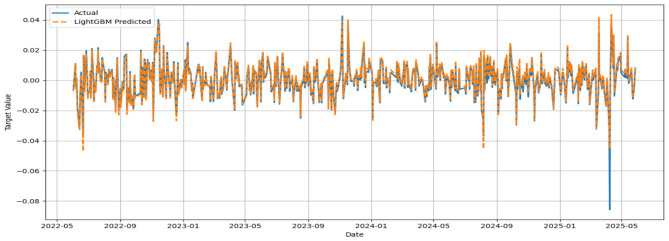
Actual and predicted figures for LGBM modeling.

**Figure 8:**
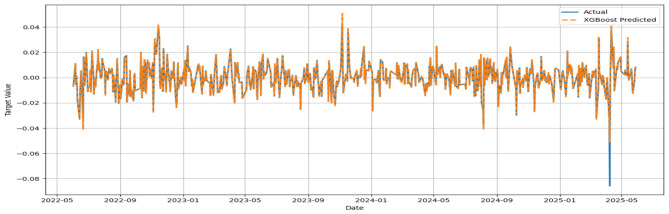
Actual and predicted figures for XGBoost modeling.

**Figure 9:**
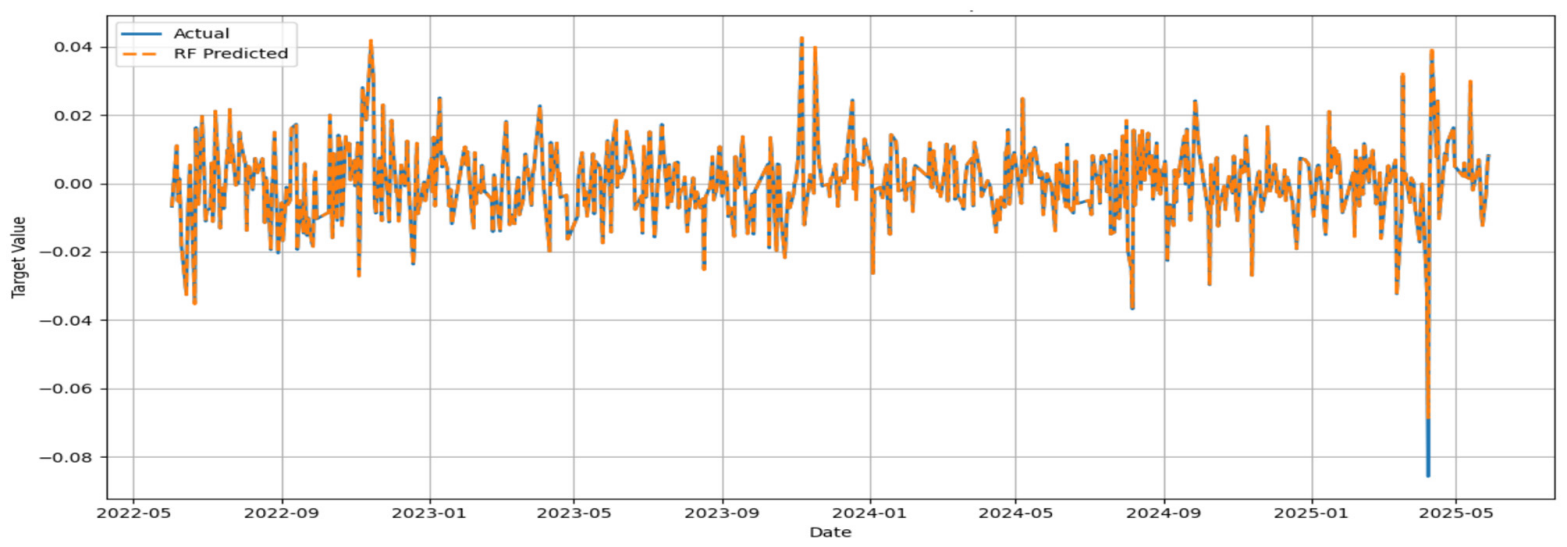
Actual and predicted figures for RF modeling.

**Figure 10:**
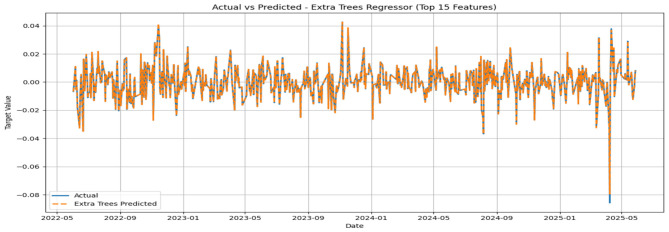
Actual and predicted figures for ETR modeling.

The MSE, RMSE, and MAE values appeared to be low, while the IA values for all models appeared to be greater than 0.7. No significant improvement in MSE could be noticed beyond 500 base learners for the respective models. In general, the convergence was reached with approximately 300 base learners. Ideally, for the prediction of daily closing prices of stock market variables, the past cognate research reports IA values above 0.85 on the test segment (Ghosh et al., 2022; Ghosh and Chaudhuri, 2022) to testify to the effectiveness of the predictive framework. However, the current attempt aims to perform predictive modeling on returns series, which closely resembles random walks. Thus, the overall efficacy of the individual models in predicting the daily returns of the NIFTY BRICS 50 series can be classified as satisfactory. Among the models, the accuracy obtained by the GBR model appears to be relatively low, while CatBoost, RF, and ETR ensemble methods fetch relatively superior predictions. Figures 5–10 visualize the predictions obtained by the respective models in contrast to the actual observations in the test data segment.

The close proximity of actual and predicted figures, barring a few highly spiked instances for all six predictive models, supplements the inferences on the effectiveness of the proposed predictive structures drawn based on the performance indicators summarized in Table 3.

### 5.3 Outcome of S.A.F.E. AI analysis

We now proceed to S.A.F.E. AI evaluation to further reveal additional insights pertinent to model explainability and performance. Initially, the robustness contributions of the 15 features identified earlier are evaluated by estimating RGR values for individual models. Table 4 enlists the findings.

**Table 4:** RGR figures of top 15 explanatory variables.

**Features**	**GBR**	**CatBoost**	**LGBM**	**XGBoost**	**RF**	**ETR**
CSI800	0.9992	0.8556	0.8796	0.8101	0.8540	0.8556
CSIFIN	0.9984	0.8563	0.8802	0.8129	0.8558	0.8563
ICON	0.9775	0.8317	0.8545	0.7898	0.8232	0.8335
IFNC	0.9928	0.8419	0.8677	0.8016	0.8401	0.8419
IMAT	0.9881	0.8380	0.8649	0.7998	0.8370	0.8380
JSE25	0.9987	0.8585	0.8807	0.8127	0.8570	0.8605
JSE40	0.8730	0.6815	0.7304	0.6681	0.6891	0.6694
LAG1	0.9978	0.8561	0.8790	0.8119	0.8551	0.8567
NMFG	0.9976	0.8603	0.8809	0.8154	0.8594	0.8634
NSERV	0.9890	0.8382	0.8693	0.7907	0.8360	0.8382
USDBRL	0.9966	0.8486	0.8739	0.8069	0.8487	0.8486
USDCNY	0.9988	0.8597	0.8823	0.8134	0.8568	0.8604
USDINR	0.9991	0.8573	0.8810	0.8124	0.8563	0.8573
USDRBL	0.9646	0.8541	0.8639	0.8111	0.8381	0.8541
USDZAR	0.9859	0.8568	0.8858	0.8131	0.8551	0.8568

It is also equally important to gauge the RGE scores at the individual level, barring the explainability impact of predictor variables, as the latter is directly responsible for driving the prediction process during volatile regimes. In addition, the RGE contribution patterns are not uniform across the models. For example, CSI800 and USDCNY emerge to be the top two critical factors responsible for robustness of the GBR model, while the said slots for the CatBoost model are occupied by NMFG and USDINR. A closer look at the table reveals that NMFG and USDCNY appear in the top five feature lists for all models. The contribution of the JSE40 in terms of augmenting the overall sustainability is substantially weakened. Therefore, it is of paramount importance to closely monitor the Indian and Chinese economies to anticipate and predict the fluctuations and unexpected movements in NIFTY BRICS 5O movements. We now compute the other two S.A.F.E AI metrics in conjunction with the aggregate RGA, RGR, and RGE scores for relative evaluation of the utilized predictive models and report the results in Table 5.

**Table 5:** Model comparison through S.A.F.E. AI lens.

**Models**	**RGA**	**RGR**	**RGE**
GBR	0.7403	0.7084	0.4599
CatBoost	0.8177	0.5392	0.4399
LGBM	0.7791	0.5950	0.4864
XGBoost	0.7786	0.5618	0.4897
RF	0.8215	0.5556	0.4848
ETR	0.8309	0.5445	0.4787

In terms of accuracy, manifested by the RGA indicator, ETR outshines the other models. The RGR metric, responsible for evaluating the capacity of models to withstand the impact of extreme data points, suggests the relative superiority of the GBR model over others. Finally, RGE figures imply the edge of XGBoost in terms of better explainability than the other models. Thus, no clear dominance is evident for the competing model in decoding the BRICS NIFTY 50 daily returns. We resort to the multi-criteria decision-making framework for ranking the models, wherein the four predictive indicators, in alliance with the three S.A.F.E AI metrics, form the criteria set, and the six predictive models act as the alternatives. Table 6 furnishes the outcome.

**Table 6:** Final model ranking.

**Model ranking framework**	**GBR**	**CatBoost**	**LGBM**	**XGBoost**	**RF**	**ETR**
**MEREC-CoCoSo**						
Preference score	1.2426	2.9005	3.0425	2.9406	3.5683	3.7171
Ranking	6	5	3	4	2	1
**MEREC-TOPSIS**						
Closeness coefficient	0.2678	0.6469	0.5341	0.5126	0.7140	0.7436
Ranking	6	3	4	5	2	1

It can be seen that in terms of aggregate performance, ETR transpires to be relatively superior, followed by RF. Nevertheless, it should be noted that the other models are also equally efficient in fetching accurate predictions and other manifestations under model explainability issues. The ETR enjoys a marginal edge over others if all aspects of accuracy and explainability are concerned. Traders and different market players can effectively leverage the ETR model for investing and risk mitigating accordingly. We have additionally explored the RGE scores of the top 15 features for two additional series transformations of daily BRICS NIFTY 50 returns using the ETR model as a predictive framework owing to its marginal superiority.

In addition, we have conducted a sensitivity analysis to validate the comparative ranking process. The present work follows the procedure of Pamucar et al. (2020), wherein the impact of the change in the weight of RGR criteria on the resultant ranking was critically delved into. A total of 20 simulations (Sim), equally distributed between the MEREC-CoCoSo and MEREC-TOPSIS frameworks, have been trialed by changing their weight coefficients. The simulation is performed based on the proportion:


(45) $$\begin{array}{l} w_{n}:\left(1-w_{RGR}\right)=w_{n}^{*}:\left(1-w_{RGR}^{*}\right) \end{array}$$

Where *w*_*n*_ represents the actual weight of the considered criterion, *w*_*RGR*_ denotes the actual weight of the criterion, RGR, $w_{n}^{*}$ reflects the corrected magnitude of the considered criterion, and $w_{RGR}^{*}$ symbolizes the corrected weight of RGR.

Tables 7, 8 delineate the outcome of the sensitivity analyses.

**Table 7:** Outcome sensitivity analysis for MEREC-CoCoSo.

**Models**	**Sim 1**	**Sim 2**	**Sim 3**	**Sim 4**	**Sim 5**	**Sim 6**	**Sim 7**	**Sim 8**	**Sim 9**	**Sim 10**
GBR	6	6	6	6	6	6	6	6	6	6
CatBoost	5	5	5	5	5	5	5	5	5	5
LGBM	3	3	3	3	3	3	3	3	3	3
XGBoost	4	4	4	4	4	4	4	4	4	4
RF	2	2	2	2	2	2	2	2	2	2
ETR	1	1	1	1	1	1	1	1	1	1

**Table 8:** Outcome sensitivity analysis for MEREC-TOPSIS.

**Models**	**Sim 1**	**Sim 2**	**Sim 3**	**Sim 4**	**Sim 5**	**Sim 6**	**Sim 7**	**Sim 8**	**Sim 9**	**Sim 10**
GBR	6	6	6	6	6	6	6	6	6	6
CatBoost	3	3	3	3	3	3	3	3	3	3
LGBM	4	4	4	4	4	4	4	4	4	4
XGBoost	5	5	5	5	5	5	5	5	5	5
RF	2	2	2	2	2	2	2	2	2	2
ETR	1	1	1	1	1	1	1	1	1	1

It can be clearly observed that the rankings remain unaltered for both methodologies across different simulations. Thus, the ranking of predictive models can be inferred to survive the sensitivity analysis, thereby emerging as robust.

## 6 Implications

The index under study is composed of shares of companies of developed countries that have exposure in the BRICS countries. This exposure can be trade related through export and import of final goods and services, trade in intermediate goods, and raw materials such as crude oil, iron ore, and coal. The relationship can also be through transfer of technology, outsourcing, licensing, or direct investments in production facilities. In any of the above, exchange rate fluctuations will affect profitability of the companies of the developed countries. In our predictive framework, if we look at the top explanatory variables, all of the five exchange rates emerge significant. This is a major result of the study. It implies that NIFTY BRICS 50, as an instrument for global financial diversification, is most sensitive to trade relations and transnational fund flows.

Production and trade can proceed efficiently under stable macroeconomic conditions, and the latter depends on the performance of the manufacturing and the services sector. India has experienced significant economic growth, and its manufacturing and service sectors have global presence. This is reflected in the significance of NMFG and NSERV. China has emerged as one of the leading nations in the world, and our study reveals the importance of the impact of industrial growth, service sector growth, and financial sector growth indicators on the index. For Brazil, the impact of the basic material sector, service sector, and financial sector growth indicators emerges as significant features. This is a reflection of their growth process. Surprisingly, our results indicate that, while for South Africa only service sector growth has an impact on the index, none of the factors for Russia impact the index significantly. This can imply that the relationship of the BRICS Index companies with Russia is purely through trade.

A practical application of the study is that while monitoring the index returns, fund managers should focus on exchange rates. Furthermore, the results also indicate that while assigning country weights to their global funds, fund managers need to closely follow the macroeconomic growth pattern of the different economies.

Fund managers are focused on monitoring returns from the funds that they manage. This helps them convince their clients to choose assets and their weights in the portfolio. The proposed AI-driven predictive has demonstrated the correctness of the choice of explanatory variables, as well as their predictive efficiency. GBR and SHAP-based XAI have been used to identify the top significant country-specific explanatory variables. This is a contribution of the study. Subsequently, with the selected variables, GBR, CatBoost, Light Gradient Boosting Machine (LGBM), XGBoost, RF, and ETR are applied for forecasting returns from BRICS NIFTY 50. Along with standard evaluation tools, AI-driven S.A.F.E framework is used for measuring predictive accuracy, sustainability, and contribution of each predictor. This is, again, a significant contribution in determining the robustness of the predictive methods chosen.

In view of the number of predictive models and efficiency indices used, MCDM models were used to rank the methods for further clarity. ETR emerged as the most efficient predictor.

## 7 Conclusion

A significant aspect of the study was to determine predictive efficiency through the S.A.F.E AI lens. While efficiency indicators such as MSE, RMSE, MAE, and IA were calculated, sustainability, accuracy, fairness, and explainability of the framework were also determined. The overall ranking of the various prediction methodologies was evaluated taking all these efficiency indicators into consideration. The prediction process introspection through the S.A.F.E AI lens offers additional insights and flexibility in comparison with SHAP-driven XAI. The former enables predictive models to rank across different dimensions, such as RGA, RGR, and RGE. The findings suggest the ranking of the utilized models across these dimensions is not uniform. Therefore, depending on the priority of stakeholders, the appropriate model can be recommended by S.A.F.E AI. Nonetheless, the final comparative examination using the MCDM-based check and applying the MEREC and CoCoSo methods in tandem ensures a nuanced comparative evaluation that considers RGA, RGR, and RGE together. The present study emerges to be practically relevant in propounding a robust architecture for decoding the governing patterns of global portfolio-type funds by fetching accurate forecasts and simultaneously uncovering the dependence structure on transnational volatility, economic growth, and trade relations. Overall, the present research has accomplished the objectives and contributes to the existing strand of literature.

The study has highlighted the importance of the proposed approach for global fund managers and has provided actionable strategic insights. It has also provided insight to policymakers that global funds move in and out of countries very fast today, putting pressure on the exchange rate, as well as the macroeconomy. To retain these funds, the role of the manufacturing, the service, and the financial sector and their growth are important. Developments in the real sectors, along with financial stability, generate confidence among global fund managers. Policymakers have to ensure that.

While the focus has been on funds returns, in future the Sharpe Ratio will be included in the analysis. The present study is limited to a single index. Our future research will focus on testing the model with other global indices to determine the generalizability of the results. It will also be interesting to introspect the empirical dynamics, reactions, and dependence of global funds during regimes characterized by extreme distress and geopolitical turmoil. Another natural progression of the current research would be to extend the methodological lens by integrating advanced deep learning models. A comparative assessment with alternative techniques such as LIME, PDP, or ALE would be attempted to strengthen the robustness of the results.

We finally mention that all codes, data, and tools used in this study will be made available upon request.

## Data Availability

The raw data supporting the conclusions of this article will be made available by the authors, without undue reservation.
